# *Campylobacter jejuni* versus *Campylobacter coli*: Occurrence and antimicrobial resistance in red meat sold in markets in Wasit, Iraq

**DOI:** 10.5455/javar.2025.l940

**Published:** 2025-06-02

**Authors:** Manal Hadi Ghaffoori Kanaan, Ahmad M. Tarek

**Affiliations:** 1 Department of Nursing, Technical Institute of Suwaria, Middle Technical University, Baghdad, Iraq.; 2 Department of Crime Evidence, Institute of Medical Technology Al-Mansour, Middle Technical University, Baghdad, Iraq.

**Keywords:** Antibiotic resistance, cattle meat, thermotolerant *Campylobacter*, sheep meat

## Abstract

**Objective:**

*Campylobacters* are common causative gastroenteritis pathogens in humans, and they are a leading cause of food poisoning globally. The present investigation sought to assess the occurrence and antibiotic resistance of *Campylobacter* species recovered from cattle and sheep meat sold in markets in Wasit, Iraq.

**Materials and Methods:**

Using conventional microbiological methods, 113 samples were collected from nearby marketplaces and tested to assess the occurrence of* Campylobacter*. *Campylobacter* species were confirmed using multiplex polymerase chain reaction. To determine the susceptibility to certain antimicrobial agents, a disk diffusion assay was carried out, and eight different antimicrobial drugs were tested.

**Results:**

The findings revealed that *Campylobacter’*s isolation rate was 10.62%, with 10.77% and 10.42% in cattle and sheep meat samples, respectively. Additionally, 75% of the bacterial isolates were identified as *Campylobacter jejuni* (*C*.* jejuni*), while 25% were confirmed as *Campylobacter coli* (*C*.* coli*). One hundred percent of bacterial isolates exhibited resistance to oxacillin, erythromycin, nalidixic acid, and cloxacillin. Moreover, an abundance of multidrug-resistant *Campylobacter* species was identified, with eight antibiotypes classified into four categories. Likewise, the bacterial isolates‘ multiple antibiotic resistance index ranged from 0.5 to 0.88.

**Conclusion:**

According to the current study, cattle and sheep meat pose a potential threat to public health. Therefore, minimizing *Campylobacter* infection and ensuring the safe use of antibiotics requires strict monitoring, regulatory measures, and suitable treatments.

## Introduction

Foodborne illness is a significant public health issue owing to its rising incidence globally and the substantial morbidity and mortality associated with bacterial infections [1]. There have been thousands of illnesses caused by foodborne bacteria, and these diseases threaten human health and the economy as a whole [2]. Various harmful microbes may contaminate food items at any point in the production, processing, storage, or transportation phases leading up to their consumption [2]. Foodborne human pathogens are estimated to cause widespread intestinal disorders, resulting in considerable financial and health burdens [3, 4]. Children under 5 years of age account for over 30% of all cases of food poisoning, according to the World Health Organization (WHO) [5]. Thermotolerant* Campylobacter *species rank among the most prevalent bacterial pathogens linked to foodborne illnesses worldwide [6]. They cause diarrhea in 400 to 500 million individuals and 37,600 fatalities globally [7].

However, their epidemiology in the Middle East is mostly unknown [7]. Since the frequency of these infections varies among regions, it is feasible that they are more common than *Shigella* and *Salmonella* in certain nations [8]. While several species can cause campylobacteriosis, *Campylobacter jejuni* and *Campylobacter coli* are the most prevalent [9]. Following campylobacteriosis, gastroenteritis, and other symptoms occur. Fever, stomach discomfort, and diarrhea, which may sometimes be associated with blood, are frequent gastrointestinal symptoms. Other issues include septicemia, reactive arthritis, Guillain-Barre, and Miller–Fisher syndromes [10]. The majority of neurological complications result from infections caused by *C*. *jejuni* [11]. Even barely detectable levels of *Campylobacter *cells in food might be harmful to humans due to the low infectious dose [12]. The infectious dose of *Campylobacter* is around 500 CFU/gm, depending upon the individual‘s age and physical condition. The virulence mechanisms of these bacteria facilitate infection by toxin production, flagellar motility, adhesion, and epithelial cell penetration [1]. Andritsos et al. [13] discovered that although chickens are responsible for the majority of campylobacteriosis cases, other animals such as sheep and cattle frequently harbor *C*.* jejuni* and *C*.* coli* as intestinal commensals. Additionally, *Campylobacter* may be acquired from infected animals, which usually carry the bacteria without symptoms [14]. Due to the fact that *Campylobacter* is not often found on carcasses or in beef and sheep meat, it is not considered to be a significant vehicle of transmission in human illnesses. Nevertheless, the epidemiology of a number of different *Campylobacter* species has been documented in red meat in various nations [15].

Antimicrobial resistance denotes the adaptation of bacteria and other pathogens that develop defense mechanisms rendering them less sensitive to antimicrobial treatments [16]. This is a global issue that results in millions of fatalities annually and is considered a significant threat to contemporary innovation, global health, and food security [17]. In May 2015, the World Health Assembly acknowledged the critical nature of this issue by approving a global strategy to combat antibiotic resistance [18]. This approach necessitates a comprehensive analysis of the global economic landscape to guide the development of a sustainable investment strategy over the long term [19]. The inappropriate administration of antimicrobial medications, in addition to their excessive use in healthcare and agriculture, has resulted in a deterioration in the clinical effectiveness of these treatments, which has been linked to a rise in fatality rates [19]. As a consequence of this, there has been a rise in the proportion of microorganisms that are resistant to these therapies. As a result of the severe effects of antibiotic resistance, it has been named the “silent pandemic,” which is related to the alarming prediction that 10 million people could die each year by the year 2050 [20]. Within the last several years, the development of resistance in *Campylobacter *spp. to antibiotics has emerged as a significant public health alarm, not only in wealthy nations but also in underdeveloped ones [21]. An increase in the frequency of *Campylobacters* that are resistant to antimicrobials has been detected on a global scale [22]. Notably, there has been evidence of increasing resistance to aminoglycosides in human and animal strains of these bacteria, along with high fluoroquinolones and macrolides resistance [23]. While antibiotic therapy is usually not needed for campylobacteriosis since the condition usually resolves on its own, it may be essential in cases when the patient has severe symptoms or a damaged immune system [10]. Over many years, fluoroquinolones, particularly ciprofloxacin, were considered to be the best therapy [24]. However, macrolides are now being suggested as the first line of therapy for humans because of high levels of resistance to these antibiotics [24].

Although there is a lack of sufficient data from Asia and the Middle East regions, current worldwide data shows that the incidence of campylobacteriosis has been rising in most countries [25]. In Wasit province, consumers prefer red meat over other food items from retail vending, restaurants, street vendors, and small shops. However, there is currently no published data on *Campylobacter *contamination of red meat. Thus, this study evaluated the occurrence and antimicrobial susceptibility of *Campylobacter* spp. in red meat from Iraqi Wasit markets, aiming to aid in the microbiological and epidemiological evaluation of this retail meat at the consumer level.

## Materials and Methods

### Ethical approval

The Medical Ethics Committee/Middle Technical University, Iraq, approved this study (MEC No. 18). No humans or animals were involved in this study. Standards were followed for all processes.

### Sample collection

From October 2022 to September 2023, 113 red meat samples (65 cattle meat and 48 sheep meat samples) were obtained from various supermarkets and supply shops. All red meat samples were transferred to the microbiology laboratory in separate ice-free containers, shielded from sunlight, and processed within three hours after collection.

### Campylobacter isolation and identification

Following previously reported procedures by Kanaan and Khashan [26], standard microbiological techniques for isolating *Campylobacter* spp. were followed according to the International Organization for Standardization (ISO) 10272-1:2017 [27]. A total of 25 gm of each sample was placed in a sterile stomacher bag. Then, 225 ml of Bolton enrichment broth (Oxoid, CM0983), which contains [Bolton broth selective supplement (Oxoid, SR0183) and *Campylobacter* growth supplement (Oxoid, SR0232E)], was added. The mixture was stomached for 2 min and incubated at 42°C for 24 h in a microaerophilic environment (5% O^2^, 10% CO^2^, 85% N^2^) inside an anaerobic jar (Oxoid, AG25). Plates of Preston agar (Oxoid, CM0689) nourished by modified Preston *Campylobacter *selective supplement (Oxoid, SR0204), *Campylobacter *growth supplement (Oxoid, SR0232), and 5% lysed horse blood (Oxoid, SR0048) were then inoculated with 20 µl of the enrichment broth and incubated in a microaerophilic environment at 42°C for 72 h. Colonies that showed the characteristic *Campylobacter* morphology (smooth-edged round to irregular, translucent white growth may become film-like transparent spreading, and some colonies look tin or slightly pink) were extracted by growing on Preston agar base (Oxoid, CM0689). Colonies were preserved in Tryptone Soya Broth (Oxoid, CM0129) along with 20% (v/v) glycerin at −18°C. Additional identification was done, including Gram stain, catalase, oxidase, nitrate reduction, sodium hippurate hydrolysis test, and various temperature growth. BioMérieux API^®^ verification kit API CAMPY (BIOMERIEUX, 20800) was used for identifying thermotolerant *Campylobacter*.

### Confirmation of bacteria

Using primers prepared by Denis et al. [28], the mPCR method was conducted to validate the presumptive colonies‘ identification at the species level. According to Table 1, three genes were used to identify *Campylobacter *spp., *C*.* jejuni*, and *C*.* coli*.

### DNA extraction

Pure stock cultures were grown in Lauryl tryptose broth (Oxoid, CM0451), and their DNA was extracted and purified following the manufacturer‘s instructions using the Wizard^®^ Genomic DNA Extraction Kit (Promega, USA).

### PCR conditions and cycle programs

The 25 μl reaction mixture in each PCR tube consisted of 5.00 μl template DNA and 20 μl master mix (Promega, USA). PCR was performed using a Perkin–Elmer thermal cycler system, with an initial denaturation at 95°C (10 min, 1 cycle) and 35 cycles composed of 30 sec of denaturation, 90 sec of annealing at 59 °C, and 1 min of extension at 72°C. Finally, a 10 min extension at 72°C was added to the last cycle. Electrophoresis on agarose gel (1.20%) was used to identify amplified PCR products. After staining with 1.00% ethidium bromide, a gel was photographed using a UV transilluminator (Alpha Imager HP, Alpha Innotech, CA, USA) for analysis. DNA molecule size was measured using a 100-bp ladder. *Campylobacter coli* and *C*.* jejuni* strains acquired from a previous investigation [26] served as positive controls and deionized water served as a negative control.

**Table 1.** Primer sequences for PCR [28].

Specificity	Gene	Size (bp)	Primer sequence
*Campylobacter *genus	*16S rRNA*	857	Forward: ATC TAA TGG CTT AAC CAT TAA AC Reverse: GGA CGG TAA CTA GTT TAG TAT T
*Campylobacter jejuni*	*mapA*	589	Forward: CTA TTT TAT TTT TGA GTG CTT GTG Reverse: GCT TTA TTT GCC ATT TGT TTT ATT A
*Campylobacter coli*	*ceuE*	462	Forward: AAT TGA AAA TTG CTC CAA CTA TG Reverse: TGA TTT TAT TAT TTG TAG CAG CG

*16S rRNA *= 16S ribosomal ribonucleic acid; *MapA* = medium adhesion protein A; *ceuE* = gene encoding an iron transport protein.

### Antibiotic susceptibility test

*Campylobacter* isolates were tested for antibiotic susceptibility via disc diffusion assay. Interpretation of results was based on Clinical and Laboratory Standards Institute guidelines [29] that guided findings interpretation. The inoculum was prepared by directly floating isolated colonies in broth [30], a method recommended for fastidious bacteria like *Campylobacter*. To test the susceptibility of isolates to vancomycin (VAN) 30 μg, gentamicin (GM) 10 μg, oxacillin (OX) 1 μg, erythromycin (E) 15 μg, tetracycline (T) 30 μg, nalidixic acid (NA) 30 μg, ofloxacin (OFL) 5 µg, and cloxacillin (CX) 5 μg, sterile swabs were used to evenly spread the inoculum on Mueller–Hinton agar (Oxoid, CM0337) with 5% lysed horse blood (Oxoid, SR0048). All Petri dishes were incubated microaerobically overnight at 42°C.

### Statistical analysis

Data analysis was performed using MedCalc Software BVBA Version 22.021 (BE, USA). The descriptive statistics were percentage, mean, and SD. A *t*-test with 5% significance was used to assess mean ± SD for selected antibiotics, while the Chi-square (χ^2^) test was used to compare percentages [31].

## Results

### Occurrence of bacteria

**Figure 1.**
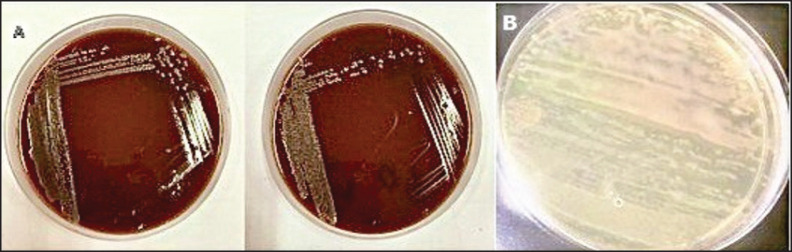
Bacterial isolation, and purification. (A) Growth on Preston agar; (B) Purification on Preston agar base without supplement.

**Figure 2.**
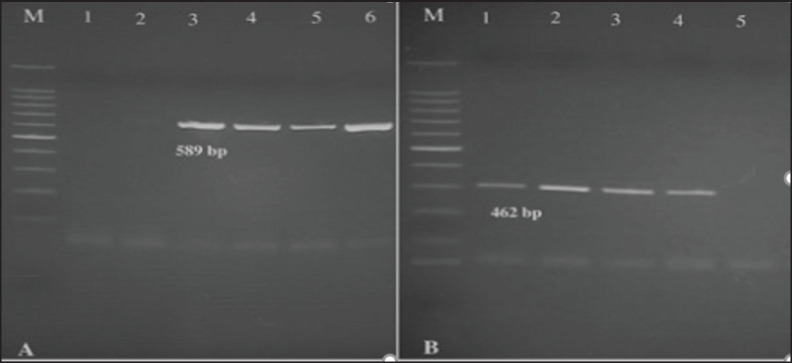
PCR results. A: M: Ladder 100 bp; Lane 1: Negative control; Lane 2: Negative sample for *Campylobacter jejuni*; Lane 3: Positive control (*C. jejuni*); Lanes 4–6: C. *jejuni isolates*. B: M: Ladder 100 bp; Lane 1: Positive control (*Campylobacter coli*); Lane 2–4: *C. coli isolate*; Lanes 5: Negative control.

The occurrence of thermotolerant *Campylobacter* in retail red meat was 10.62%, with 10.77% and 10.42% isolation rates in cattle and sheep meat, respectively (Table 2). *Campylobacter jejuni* and *C*.* coli* made up 75% and 25% of 12 presumptive isolates verified as *Campylobacter* spp. by mPCR (Table 2, Figs. 1, 2). Moreover, sheep meat had the greatest incidence of *C*.* jejuni* (80%), while cattle meat had the greatest isolation rate of *C*.* coli* (28.6%). The sample type did not significantly affect *Campylobacter* occurrence (*p* ≥ 0.05). Additionally, bacterial species significantly affected (*p* ≤ 0.05) the occurrence of *Campylobacter* (χ^2^ = 5.750, *p *= 0.0165).

**Table 2.** Occurrence of *Campylobacter *species in retail red meat.

Sample type	Number of samples	Positive samples with *Campylobacter* n/N (%)	Positive samples with *Campylobacter jejune* n/N (%)	Positive samples with *Campylobacter coli* n/N (%)
Cattle meat	65	7/65 (10.77)	5/7 (71.4)	2/7 (28.6)
Sheep meat	48	5/48 (10.42)	4/5 (80)	1/5 (20)
Total	113	12/113 (10.62)	9/12 (75)	3/12 (25)
*p *value		0.9526	0.7454	0.7454

### Antibiotic resistance

**Figure 3.**
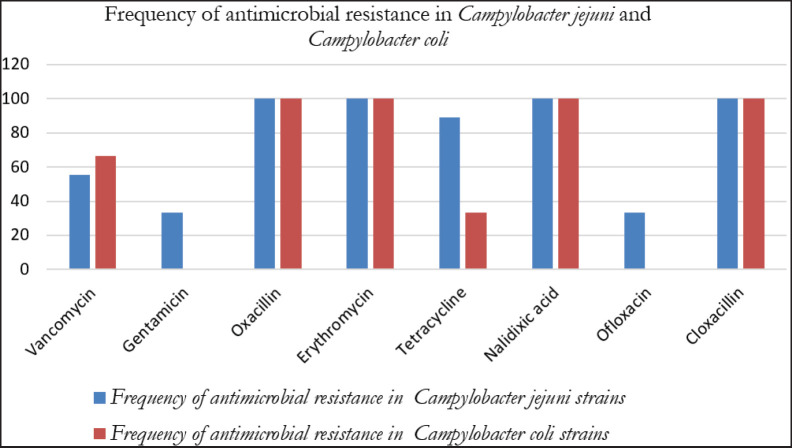
Prevalence of antimicrobial resistance in *Campylobacter jejuni* and *Campylobacter coli* recovered from red meat samples.

**Figure 4.**
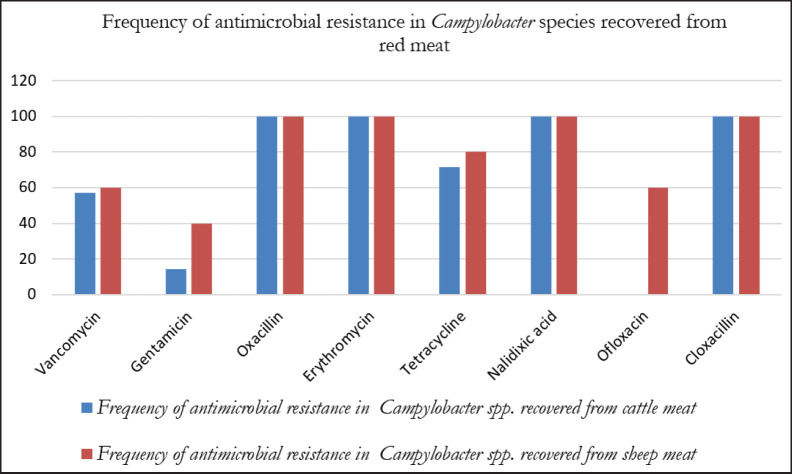
Prevalence of antimicrobial resistance in *Campylobacter* strains recovered from red meat samples.

All bacterial strains (100%) were resistant to OX, E, NA, and CX; 75% to T; 58.3% to VAN; and 25% to GM and OFL (Table 3). Resistance to GM and OFL was detected only in *C*.* jejuni* (Fig. 3). Furthermore, 50% and 75% of *C*.* jejuni* from sheep meat exhibited resistance to GM and OFL, respectively. No resistance to OFL was found in *C*.* jejuni* recovered from cattle meat, although one strain (20%) was GM resistant (Fig. 4). The study found no significant differences (*p *≥ 0.05) in resistance levels among organisms’ species; however, a significant difference (*p *≤ 0.05) in inhibitory zones for OFL was observed (*t* = −2.992,* p* = 0.0135), according to sample type and resistant bacteria (Table 4).

The bacterial resistance patterns (ARPs) and MAR index are shown in Table 5. Our study revealed that every recovered strain was resistant to at least four different antibiotics. Based on the number of antibiotics that are resisted by each strain, we classified them into four different antibiogroups. Furthermore, 33.33% of strains exhibited resistance to seven antibiotics with three different antibiotypes (CX OX VAN T NA GM E), (CX OX T NA OFL GM E), and (CX OX VAN T NA OFL E).

Further analysis revealed that 83.33% of the tested bacteria exhibited MDR against at least five drugs, with 66.66% of strains displaying resistance against at least five antimicrobials. Moreover, the incidence of MDR *Campylobacter *strains against seven agents is higher in sheep meat strains (*n =* 3, 25%). In addition, the frequency of *Campylobacter* strains that were detected with a MAR index of 0.5, 0.63, 0.75, and 0.88 was 16.7%, 33.33%, 16.7%, and 33.33%, respectively, as shown in Table 5.

**Table 3.** Antibiotic resistance in *Campylobacter* species isolated from retail red meat

Antibiotic group	Antibiotics	No. (%) of resistant isolates					
		Sample type					
		Cattle meat (7)		Sheep meat (5)		Total (n=12)	*p* value
		*C. jejune* (*n =* 5)	*C. coli* (*n =* 2)	*C. jejune* (*n =* 4)	*C. coli* (*n =* 1)		
Glycopeptide	Vancomycin	3 (60)	1 (50)	2 (50)	1 (100)	7 (58.3)	0.7462
Aminoglycosides	Gentamicin	1 (20)	0 (0)	2 (50)	0 (0)	3 (25)	0.2690
Beta-lactam	Oxacillin	5 (100)	2 (100)	4 (100)	1 (100)	12 (100)	0.9996
	Cloxacillin	5 (100)	2 (100)	4 (100)	1 (100)	12 (100)	0.9996
Macrolides	Erythromycin	5 (100)	2 (100)	4 (100)	1 (100)	12 (100)	0.9996
Tetracyclines	Tetracycline	5 (100)	0 (0)	3 (75)	1 (100)	9 (75)	0.0654
Quinolones and Fluoroquinolones	Nalidixic acid	5 (100)	2 (100)	4 (100)	1 (100)	12 (100)	0.9996
	Ofloxacin	0 (0)	0 (0)	3 (75)	0 (0)	3 (25)	0.2690

*Campylobacter jejuni *= *C. jejuni, Campylobacter coli* *= C. coli*

## Discussion

Human campylobacteriosis is linked to undercooked meat contamination with these bacteria. Contamination of food with thermotolerant *Campylobacter* spp. can occur at all stages of the food supply chain, including production, processing, distribution, and preparation [30].

Several studies have been conducted on *Campylobacter *in chicken meat samples in Iraq and worldwide, but few have focused on cattle and sheep meat. The occurrence of thermotolerant *Campylobacter *in retail red meat was 10.62% (Table 2), with 10.77% and 10.42% in cattle and sheep meat, respectively. Similar results were found in Iran and Poland [32, 33]. Feces from slaughtering may contaminate meat, posing a *Campylobacter* risk [34]. Foods derived from animals have been implicated as the primary agents responsible for the transmission of *Campylobacter* infection in humans [5]. Given that raw meat from cattle and sheep is consumed in large quantities in Iraq, the presence of *Campylobacter* in meat and meat products increases the probability that the pathogens will be transmitted to people. Previous research found a lower occurrence of thermotolerant *Campylobacter* compared to our findings [24, 35, 36]. Likewise, Berhanu et al. [15] also reported a lower isolation percentage of thermotolerant *Campylobacter* (7.9%) than that obtained in our study. These bacteria may tolerate thermal stress in food during storage due to increased preliminary microbial counts and chromosomal differences among isolates [30], which may explain its higher frequency in our study. A higher incidence than our findings was reported in Malaysia and Iran [37, 38].

**Table 4.** Influence of sample source on antibiotic resistance in *Campylobacter* species.

Antimicrobial agents	Origin of isolates				
	Cattle meat		Sheep meat		*p* value
	Zones’ inhibition (mm)	Mean ± SD	Zones’ inhibition (mm)	Mean ± SD	
Vancomycin	0–16	5.86 ± 6.98	0–13	4.6 ± 5.71	0.7480 NS
Gentamicin	10–21	15.57 ± 3.58	8–19	14 ± 3.74	0.4788 NS
Oxacillin	0–10	1.43 ± 3.5	0–10	2 ± 4	0.7982 NS
Erythromycin	0–1	0.14 ± 0.35	0–11	2.2 ± 4.4	0.2369 NS
Tetracycline	10–22	14.1 ± 5.1	12–19	14.6 ± 2.4	0.8441 NS
Nalidixic acid	0–14	5.3 ± 6.2	0–10	4 ± 4.9	0.7058 NS
Ofloxacin	13–15	14 ± 0.93	10–14	12 ± 1.4	0.0135 S
Cloxacillin	0–8	1.14 ± 2.8	0–10	0.2 ± 0.4	0.4791 NS

NS= no significant; S= Significant; SD= Standard deviation.

Various data suggest that time, season, sampling location, and laboratory methods may affect prevalence estimates [37, 38]. According to our results, the majority of the *Campylobacter* species found in our samples belonged to *C*. *jejuni*, which aligns with the results previously reported [15, 24, 37–39]. It is worth noting that the manual nature of the slaughtering, evisceration, and skinning processes at Wasit abattoirs poses the risk of cross-contamination. Therefore, the sanitation procedure used in slaughterhouses may effectively reduce or eliminate contaminating bacteria.

*Campylobacter* species tend to be developing resistance to therapeutically essential antibiotics, threatening public health. Many nations have reported high frequencies of resistance to quinolones, fluoroquinolones, and T, whereas *C*.* jejuni* resistance to E and GM is limited [40]. Interestingly, all isolates were resistant to OX, E, NA, and CX in our investigation (Table 3). Additionally, T and VAN resistance was 75% and 58.3%, respectively, which was greater than prior studies [24, 37, 38]. A prior Ethiopian study [15] documented high resistance rates for beta-lactams in *C*.* jejuni*, ranging from 81.8% to 100%, while *C*.* coli* exhibited beta-lactam resistance between 66.7% and 100%, with a lower resistance rate of 33.3% to E. This contrasts with the findings of the current study, where all isolates demonstrated resistance to E, raising considerable concerns as it restricts treatment options for *Campylobacter* infections.

**Table 5.** Antibiogram of *Campylobacter* species from retail red meat.

Number of antimicrobials used/antibiotype	Antibiotypes	Sample type				Antibiogroups	Total 12 (%)	MDR Index
		Cattle meat (7)		Sheep meat (5)				
		*C. jejuni* 5 (%)	*C. coli* 2 (%)	*C. jejuni* 4 (%)	*C. coli* 1 (%)			
8/7	CX OX VAN T NA GM E	1 (20)	0 (0)	1 (25)	0 (0)	1A	4 (33.33)	0.88
8/7	CX OX T NA OFL GM E	0 (0)	0 (0)	1 (25)	0 (0)	1B		
8/7	CX OX VAN T NA OFL E	0 (0)	0 (0)	1 (25)	0 (0)	1C		
8/6	CX OX VAN T NA E	2 (40)	0 (0)	0 (0)	0 (0)	2A	2 (16.7)	0.75
8/5	CX OX T NA E	2 (40)	0 (0)	0 (0)	0 (0)	3A	4 (33.33)	0.63
8/5	CX OX VAN NA E	0 (0)	1 (50)	0 (0)	0 (0)	3B		
8/5	CX OX NA OFL E	0 (0)	0 (0)	1 (25)	0 (0)	3C		
8/4	CX OX NA E	0 (0)	1 (50)	0 (0)	1 (100)	4A	2 (16.7)	0.5
	Total	7/7 (100)		5/5 (100)		4	12 (100)	

CX = Cloxacillin; OX = Oxacillin; VAN = Vancomycin; T = Tetracycline; NA = Nalidixic acid; GM = Gentamicin; E = Erythromycin; OFL = Ofloxacin.

In an Italian investigation, most *Campylobacter* strains had 81.45% NA resistance [24]. Other research in Malaysia, Korea, and Tanzania found significant T resistance in *Campylobacter *isolates [37, 41, 42].

There are many different reservoirs for antibiotic resistance genes, including bacteria, people, animals, water, and the environment. These genes may be passed on from one reservoir to another. The proportional importance of several transmission pathways varies across bacterial species and resistance genes [43].* Campylobacter*’s innate resistance to numerous beta-lactam medicines renders its usage unfavorable, particularly in severe infections [44]. E resistance may be linked to the widespread use of spiramycin to treat and manage bacterial and mycoplasma illnesses in cattle and poultry [45]. Untreated chicken manure fertilizer may contribute to *Campylobacter *isolates’ high quinolone resistance [44]. High VAN resistance in *Campylobacter* isolates indicates intrinsic resistance [45]. Tet(O) plasmid caused most *Campylobacter* T resistance, and 60%–100% of *C*. *jejuni* and *C*. *coli* carried T resistance plasmids [46]. *Enterococci* spp., MDR gut bacteria, may provide *Campylobacter* tolerance to several antibiotics by carrying many resistance genes [16, 47]. Our investigation found significant *T* and beta-lactam resistance, which may be linked to their widespread use in people and animals.

Our study found low GM resistance (Table 3), which is consistent with previous reports from Iraq and Malaysia [26, 37]. However, GM resistance was previously modest (0%–2%) [44, 48]. Conversely, *C*. *jejuni* presented more resistance to GM than *C*. *coli*, contradicting prior findings [24, 37]. Apramycin has been frequently used in veterinary therapies, which may be linked to *Campylobacter* GM resistance [34].

Antibiotic resistance patterns vary according to sample type, sampling process, antibiotic type, and frequency in animal husbandry and human medicine [37, 49].

MDR has been defined as resistance to at least three dissimilar antimicrobials found in 100% of *Campylobacter* strains (Table 5), which is higher than previous investigations [24, 37]. Other studies have shown resistance to two or more antibiotic classes [44]. The acquisition of one or more resistance determinants on similar DNA particles, like multidrug pumps that stipulate efflux activity to several medications, might cause multi-resistance [50, 51]. Genetic resistance may be chromosomal or plasmid-borne, combining endogenous and picked-up genes [52–54]. *Campylobacter* strains from sheep meat, which Iraq consumes most frequently, had higher rates of MDR with seven antibiotics. Such meat strains could affect public health and health promotion strategies.

According to Table 5, all *Campylobacter* isolates in our investigation had a MAR value of 0.5 or above. Bacteria with a MAR index above 0.2 are assumed to come from higher contamination livestock [39].

Iraq lacks antibiotic use data in animal farming. Therefore, we sought to provide valuable data to evaluate the relationship between livestock production using antimicrobial drugs and the rise of foodborne bacterial resistance, such as *Campylobacter*. Iraqi investigations on MDR in numerous foodborne pathogens supported this association [23, 34, 55–57]. Other Iraqi research also highlighted antibiotic overuse and misuse, which worsened this issue [55, 58, 59]. These findings draw attention to the need to reduce antibiotic application to minimize MDR *Campylobacters*.

The present work has certain limitations due to a lack of funding. The samples were collected from a single province, and the investigation focused only on cattle and sheep meat, excluding other red meat products. The frequency of resistance genes was not examined. However, this study represents the first investigation into the prevalence, antibiotic resistance, and antibiogram of *Campylobacter *species found in cattle and sheep meat from Iraq. Further in-depth research is recommended to be conducted in the future, spanning multiple provinces, to analyze the specific pathways that lead to antibiotic resistance in bacterial isolates from diverse foods in Iraq.

## Conclusion

This study indicates that *Campylobacter* in red meat in Iraq may pose significant public health concerns. Consuming these meats may lead to campylobacteriosis. *Campylobacter* species exhibited complete resistance (100%) to OX, E, NA, and CX, with MDR to at least three antimicrobials observed in 100% of the strains. It is necessary to implement strict sanitary standards to control the presence of antibiotic-resistant microorganisms in meat and other products. The reduction of *Campylobacter* infections may be facilitated by the enforcement of rigorous regulations on the cleanliness of slaughterhouses, as well as the prescription and administration of medications, accompanied by the establishment and implementation of health education programs. Therefore, antimicrobials must be used judiciously in both veterinary and human therapeutic protocols, and resistance patterns should be carefully analyzed for targeted application. Further research is necessary to assess the prevalence of zoonotic enteric campylobacteriosis in humans, red meat, and other animals in various study areas. A more comprehensive epidemiological investigation is needed to evaluate the role of livestock as reservoirs for this disease.

## List of abbreviations

°C, degree Celsius; *16S rRNA*, 16S ribosomal ribonucleic acid; API CAMPY, analytical profile index campy; bp, base pairs; ceuE, gene encoding an iron transport protein; CLSI, clinical and laboratory standards institute; CX, cloxacillin; DNA, deoxyribonucleic acid; DW, deionized water; E, erythromycin; gm, gram; GM, gentamicin; ISO, international organization for standardization; MapA, medium adhesion protein A; MAR, multiple antibiotic resistance; MDR, multidrug-resistant; ml, milliliter; mPCR, multiplex polymerase chain reaction; NA, nalidixic acid; OFL, ofloxacin; OX, oxacillin; SD, Standard deviation; T, tetracycline; UV, ultra-violet; VAN, vancomycin; μg, Microgram; μl, microliter.
